# Anti-Plague Inoculation

**Published:** 1900-03-03

**Authors:** 


### ANTI-PLAGUE INOCULATION.

The report of the Indian Plague Commission, so far as concerns the question of anti-plague inoculation, has now been given to the world. The report begins with a general historical survey of the subject of preventive inoculation, a survey which is sufficient to show on how broad a basis this proceeding now stands. Nevertheless, it is clear that much yet remains to be done before we can say with anything like assurance that we have in Haffkine's inoculation a satisfactory and reliable prophylactic against plague. The Commissioners criticise somewhat severely the methods adopted in the preparation of the vaccine, especially in regard to their uncertainty in producing an aseptic product, and they show that, as a fact, a number of the samples tested gave evidence of contamination of the prophylactic. Then they are very severe upon the methods of standardisation which seem to have been employed, and when one reads the details one can hardly say that they are too severe in their condemnation. It had been understood that the vaccine as sent out had been so standardised that a given volume would, on the average, produce a given result, but the Commissioners elicited that "the routine practice which was adopted in Mr. Haffkine's laboratory was to standardise the vaccine by holding up to the light one or two sample bottles of each brew with a view to appreciating the opacity of the vaccinating fluid. It was in conformity with the results of this appreciation that the dose was inscribed on the label of each bottle. This standard dose was an arbitrary quantity." Of course this does not affect the principle, but it tends, as do other things pointed out by the Commissioners, to throw doubt upon the exactitude of the experiments on which so much is being made to depend. Taking the results of the experiments as they stand, however, the Commissioners come to the conclusion that inoculation sensibly diminishes the incidence of attacks on the inoculated population. But the protection afforded is not absolute; indeed, plague has attacked persons who have undergone inoculation as many as four times in the course of two years previous to their attack, and as many as 8 per cent. of the inoculated population may suffer from plague, as was the case in Bulsar. It is thus impossible to give a numerical expression to the protection afforded by inoculation. They also say that inoculation diminishes the death-rate among those inoculated, not merely from the attack-rate being diminished, but from the fatality of the attacks being lessened. As for the duration of such protection as is conferred, this, it is held, certainly lasts for a considerable number of weeks. On the general question, then, they recommend that under the safeguards and conditions of accurate standardisation and complete sterilisation of the vaccine, and the thorough sterilisation of the syringe in each case, inoculations should be encouraged wherever possible, and particularly among disinfecting staffs and the attendants of plague hospitals. Reading between the lines of this very modified praise and this very mitigated recommendation, one can see that the Commissioners are by no means embued with any superabounding faith in the proceeding as hitherto carried out, true as the principles may be upon which it is founded.

